# Evaluating urea-based nitrogen fertilization practices on greenhouse tomato growth and nitrate leaching in cold regions

**DOI:** 10.3389/fpls.2026.1925562

**Published:** 2026-10-01

**Authors:** Changqing Li, Ziyi Wu, Haixu Wang, Shuting Li, Xiaojing Wang, Jiqing Wang, Baoyue Zhang

**Affiliations:** 1 Institute of Sericulture, Chengde Medical University, Chengde, China; 2 College of Agriculture and Forestry Sciences, Hebei North University, Zhangjiakou, China; 3 College of Economics and Management, Hebei North University, Zhangjiakou, China

**Keywords:** cold region, greenhouse tomato, nitrate leaching, quality, yield

## Abstract

**Introduction:**

There are serious problems in agricultural production, such as low nitrogen utilization rate and nitrate leaching. This study examined how different fertilization practices affect the yield, quality, and soil nitrate leaching of greenhouse tomatoes in the cold regions. The goal was to provide technical support for simple, high-quality, and efficient greenhouse vegetable production in these areas.

**Methods:**

Under integrated water-fertilization conditions in the cold regions, six fertilization treatments were applied: No nitrogen fertilizer, Conventional farmer practice (360 kg ha^-1^ N), Optimized nitrogen application (240 kg ha^-1^ N), Slow-release urea A mixed with conventional urea at 7:3 (240 kg ha^-1^ N), Slow-release urea B mixed with conventional urea at 7:3 (240 kg ha^-1^ N), Conventional urea mixed with modified slow-release polymer material (240 kg ha^-1^ N).

**Results:**

The results showed that optimized nitrogen treatments improved plant height and SPAD values, which helped increase yield. Among them, conventional urea mixed with modified slow-release polymer material had the highest yield. Optimized fertilization treatments also improved quality. Agronomic efficiency and partial factor productivity under optimized nitrogen treatments were 59.08%-231.95% higher than under conventional farmer practice. These treatments also reduced nitrate accumulation in the 60-100 cm soil layer. Using a multi-objective evaluation model based on fuzzy mathematics membership function, conventional urea mixed with modified slow-release polymer material was identified as the best option.

**Discussion:**

In summary, under integrated water-fertilization conditions, applying conventional urea mixed with modified slow-release polymer material can maintain tomato yield and quality while reducing soil nitrate leaching. This method meets the demands of modern agriculture and environmental protection.

## 1 Introduction

Tomato (*Solanum lycopersicum* L.) is the most widely cultivated greenhouse vegetable in the world. It is highly valued for its balanced sweet-sour taste and rich nutritional composition (Padayachee et al., 2017). Tomatoes were introduced to China long ago and have since become a major vegetable crop in the country. They also gradually gained importance in global trade and agricultural production (Li et al., 2016). However, with continuous cultivation, many growers have overused nitrogen fertilizers to increase yield and profit. This practice disrupts the balance between nitrogen supply and demand. It also lowers nitrogen use efficiency (NUE). As a result, the positive effect of nitrogen fertilizer on yield decreases, vegetable quality declines, and serious environmental pollution occurs through various pathways (Lin et al., 2025; Sha et al., 2020). In recent years, researchers worldwide have focused on improving NUE by modifying fertilizer release patterns, such as using urease inhibitors, nitrification inhibitors, and coated urea. Controlled-release fertilizers can prolong nutrient availability by delaying and controlling nutrient release (Liu et al., 2023; Fertahi et al., 2021; Rop et al., 2018; Jariwala et al., 2022). This ensures a continuous nutrient supply throughout the crop growth cycle. It helps increase both crop yield and quality while improving fertilizer use efficiency (Govil et al., 2024; Vejan et al., 2021). However, the nutrient release rate of some controlled-release fertilizers is influenced by soil temperature. In cold areas, such as the low-temperature regions of northwestern Hebei Province, China, this release is significantly limited. This restriction hinders crop growth and development (Ransom et al., 2020; Lin et al., 2021). Therefore, optimizing nitrogen fertilizer application technology has become an important measure, which could improve nitrogen agronomic efficiency (NAE), reduce nitrogen pollution, and support green agricultural development.

The integration of water and fertilizer technology is widely used in facility-based production, significantly improving both production efficiency and modernization. However, the supporting techniques and fertilizer products for this integrated approach remain underdeveloped. Currently, large-scale planting systems mainly rely on conventional solid compound fertilizers for drip irrigation. Yet, these solid fertilizers dissolve slowly, which often leads to uneven nutrient distribution and clogging of irrigation systems. As a result, crop growth becomes inconsistent, product quality declines, and farmers’ income is affected (Rathore et al., 2021). Although highly water-soluble fertilizers are available, their high cost makes them less attractive to farmers. Liquid fertilizer, which contains one or more essential plant nutrients, disperses easily in water. It serves as an efficient carrier in water-fertilizer integration systems. Using liquid fertilizer avoids the risks of poor dissolution and clogging associated with solid fertilizers. It also allows rapid and uniform distribution within the drip irrigation system, improving fertilization efficiency. As a soil cementing agent, polymers can enhance soil water-holding capacity (Xing et al., 2026) and have been widely adopted in water-saving agriculture and ecological restoration. Numerous studies indicate that polymer water-retaining materials can reduce soil water permeability and bulk density (Chang et al., 2021). They also improve soil aggregate structure (Tian et al., 2019). Additionally, these materials enhance the adsorption of nitrogen and phosphorus. As a result, they help increase plant dry matter accumulation and yield. These findings show that polymer water-retaining materials improve water use efficiency and soil physicochemical properties, creating a favorable soil environment for crop growth. Since the 1990s, polyacrylamide (PAM) has been recognized as an effective material for reducing soil erosion. It enhances cohesion among soil particles and maintains soil structural stability (Lee et al., 2010; Wu et al., 2011). PAM modifies soil physical, chemical, and biological properties, promotes root penetration, and mitigates wind and water erosion (Sojka et al., 2007). Currently, most studied polymer materials are in solid form (Awad et al., 2012; Lu et al., 2016; Luo et al., 2015), which limits their application in drip irrigation systems. Therefore, modifying and optimizing polymer materials is crucial for advancing facility agriculture in China.

Previous studies have extensively investigated slow release fertilizer and nitrogen management for greenhouse tomato. Nevertheless, most relevant studies were conducted under relatively warm conditions (Li et al., 2017). Conventional slow release fertilizers suffer from temperature dependent nutrient release, which limits their performance in cold solar greenhouses (Priya et al., 2024). Moreover, most reported polymer amendments are solid-form products and cannot be well adapted to drip integrated fertigation systems (Lu et al., 2016). To date, few studies have systematically evaluated whether combined application of conventional urea and modified slow release polymer can synchronously improve tomato yield and quality, enhance nitrogen use efficiency and mitigate nitrate leaching risk under cold region greenhouse fertigation. To fill this knowledge gap, the present study was performed. We hypothesized that optimized nitrogen fertilization coupled with modified polymer material could promote tomato growth and reduce nitrate leaching risk in cold region (The term “cold regions” in this study refers to the regional cold climatic background of northwest Hebei Province, characterized by low annual mean temperature and low early-spring soil temperature inside solar greenhouses, even though daytime air temperature within greenhouses can reach 22-30 °C.) greenhouses. The objectives of this study are as follows: (1) assessing the influence of the optimized nitrogen treatment on the yield and quality of greenhouse tomato in these cold regions; (2) elucidating the effects of optimized nitrogen treatment on nitrogen use efficiency, and nitrate leaching. The findings of this study will offer vital theoretical insights and practical guidance for enhancing yield and quality, promoting efficient resource utilization, and fostering the sustainable development of the agriculture in these specified regions.

## 2 Materials and methods

### 2.1 Experimental site

The experiment was carried out in a solar greenhouse at the experimental base of the Department of Horticulture, Hebei North University, located in Zhangjiakou City, Hebei Province, China (40°40’N, 114°55’E). The greenhouse was oriented north-south, with a length of 50 m, a span of 7.5 m, and a ridge height of 3 m. This region has a temperate semi-arid continental monsoon climate. The average annual precipitation is 400 mm, and the frost-free period lasts 140 days. The annual sunshine duration is 2881 hours, and the mean annual temperature is 7.7 °C. During the experiment, the microclimate in the greenhouse is as follows: the daytime temperature is 22-30 °C, the nighttime temperature is 9-16 °C, and the average temperature in the growing season is 17.2 °C. The air relative humidity is 55%-88%, and the average relative humidity in the growing season is 71%. The tested soil was classified as chestnut soil. In the surface layer (0–20 cm), the organic matter content was 23.1 g kg^-1^, total nitrogen was 1.27 g kg^-1^, and alkali-hydrolyzable nitrogen was 51.1 mg kg^-1^. The available phosphorus content was 30.4 mg kg^-1^, and available potassium was 289.1 mg kg^-1^. The soil pH was 8.09. Soil bulk density was 1.28, 1.38, 1.34, 1.40 and 1.42 g cm^-^³ at depths of 0-20, 20-40, 40-60, 60–80 and 80–100 cm, respectively.

The tomato variety used in the test was Hezuo 918. The fertilizers included urea (46% N), superphosphate (12% P_2_O_5_), and potassium sulfate (50% K_2_O), all of which are commercially available. Two types of slow-release urea were also tested: Slow-release urea A (41% N, 90-day release period), produced by Beijing Slow and Controlled Release Engineering Center; Slow-release urea B (41% N, 90-day release period), produced by Anhui Maoshi Agricultural Science and Technology Co., Ltd. In addition, a modified slow-release polymer material was used. It was synthesized from polyacrylamide, polyvinyl alcohol, and manganese sulfate. The active ingredient content in the solution was 2%. This material was developed by our research group and has been patented (Patent No. CN105801297). It improved soil aggregate water-stability and reduced aggregate destruction, increasing the proportion of soil organic carbon that can be easily oxidized (Tian et al., 2019, 2020).

### 2.2 Experimental design

Under integrated water-fertilization conditions in the cold regions, six treatments were arranged in a completely randomized design and repeated in triplicate, which included: No nitrogen fertilizer (CK), Conventional farmer practice (CF, 360 kg ha^-1^ N), Optimized nitrogen application (OPN, 240 kg ha^-1^ N), Slow-release urea A mixed with conventional urea at 7:3 (SRUA+U, 240 kg ha^-1^ N), Slow-release urea B mixed with conventional urea at 7:3 (SRUB+U, 240 kg ha^-1^ N), Conventional urea mixed with modified slow-release polymer material (MSRP+U, 240 kg ha^-1^ N). The 7:3 ratio is based on preliminary test results (Li et al., 2024). Each plot measured 3.0 m × 3.0 m (9.0 m²). There was a 0.5 m corridor between adjacent plots. Each plot was surrounded by cement walls extending to a depth of 1 m. Fertilizers were applied based on the plot area. In CF and OPN treatment, nitrogen was applied as urea in two forms: basal fertilizer and three topdressings. Topdressing was done via drip irrigation at 45, 75, and 105 days after planting. The basal fertilizer accounted for 1/4 of the total nitrogen, and each topdressing also accounted for 1/4. In T4, T5, and T6, all nitrogen was applied as a one-time basal fertilizer. All treatments received calcium superphosphate as basal fertilizer at a total rate of 120 kg ha^-1^. Potassium was applied as potassium sulfate, split into basal fertilizer and three topdressings. The timing and method of potassium application matched those of nitrogen in CF and OPN treatment. The basal potassium accounted for 1/4 of the total, and each topdressing also accounted for 1/4. The total potassium applied was 300 kg ha^-1^.

Tomato seedlings were planted on 1st May 2019 at a density of 50,000 plants ha^-1^. The seedlings were removed on 1st October 2019. A Venturi fertigation system was used for drip irrigation and fertilization. The drip irrigation pipe had a diameter of 12.0 mm and a wall thickness of 0.6 mm. A water meter was installed at the head of each treatment plot to control the irrigation amount accurately. Each plot received the same volume of irrigation water. Based on the water requirement pattern of tomatoes, the entropy-based supplemental irrigation method was applied. The irrigation thresholds were set at 70% and 100% of the field water capacity in the 0–20 cm soil layer. The total irrigation amount per plot was 300 mm (based on local production habits). Irrigation was applied on the day of planting and on the 20th, 45th, 60th, 75th, 90th, 105th, and 120th days after planting. Other management practices, such as weeding and pesticide application, followed local conventional methods.

### 2.3 Sample collection and measurement

#### 2.3.1 Measurement of agronomic traits and quality

On the 35th day after transplantation, five plants with uniform growth were randomly selected from each plot. Plant height was measured with a meter scale. It was taken as the natural height from the base of the stem to the top growth point. Stem diameter was measured at the base using a vernier calliper. Leaf chlorophyll content was determined with a SPAD chlorophyll meter (SPAD-502). Measurements were taken on the third fully expanded leaf from the top growth point. On the 40th day after transplanting, nine fruits were collected from each plot, and a quarter of each fruit was pooled to form one biological replicate, with a total of three biological replicates. Vitamin C (Vc) content was measured by molybdenum blue colorimetry. Soluble solids were determined with a refractometer. Titratable acid content was measured by titration. Soluble sugar content was determined using the anthrone method (Gao, 2006).

#### 2.3.2 Measurement of tomato yield

From the first harvest until the end of the harvest period, only fully ripe (red-ripe stage) tomato fruits were manually harvested from each plot. Deformed, rotten and diseased fruits were discarded. Harvesting was performed at 7 day intervals, with a total of 8 independent harvesting events during the growing season. The fresh weight of marketable ripe fruits was recorded as plot yield, which was further converted into yield per hectare.

#### 2.3.3 Collection and analysis of soil samples

Soil samples were collected from each plot during the flowering stage (25th day after transplanting), the first fruiting stage (35th day after transplanting), the third fruiting stage (45th day after transplanting), the fifth fruiting stage (65th day after transplanting), and the harvest stage (95th day after transplanting). Sampling followed an S-shaped pattern with five points per plot. Samples were taken at depths of 0–100 cm, divided into five layers each 20 cm thick. Soils from the same layer were mixed together and bagged. Fresh soil samples were directly used to measure nitrate nitrogen content via ultraviolet spectrophotometry. At the same time, soil bulk density in different soil layers was determined using the ring knife method. These measurements were used to calculate nitrate nitrogen accumulation in the soil profile. Soil samples from the 0–20 cm layer were air-dried. Then, we analyzed them for soil organic matter by the potassium dichromate external heating method, alkali-hydrolyzable nitrogen by the alkali diffusion method, available phosphorus by the NaHCO_3_ leaching-molybdenum-antimony colorimetric method, and available potassium by the ammonium acetate leaching-flame photometric method (Bao, 2000).

### 2.4 Statistical analysis and relevant calculation

Each treatment was performed with three biological replicates. Before statistical comparison, Shapiro-Wilk test and Levene’s test were applied to evaluate normality and homogeneity of variance, respectively. Datasets that met the assumptions of normal distribution and homogeneity of variance were analyzed using parametric statistical methods. The significance among treatments was analyzed by analysis of variance (ANOVA) using the SPSS 13.0 software (SPSS Inc., Chicago, IL, USA). For multiple comparisons, we used Least Significant Difference (LSD) at the 5% level. Data analysis in this study was conducted using Microsoft Excel for computation and OriginPro 2022 for graphical representation. The relevant indicators are calculated as follows:


$$\text{Nitrogen agronomic efficiency }\left({\text{NAE kg kg}}^{-1}\right)\;=\;\left(\text{yield in nitrogen application area}-\text{yield in control area}\right)/\text{nitrogen application rate}$$

$$\text{Nitrogen fertilizer partial productivity }\left({\text{PFPN kg kg}}^{-1}\right)=\text{ yield in nitrogen application area}/\text{nitrogen application rate}$$

$$\text{Soil nitrate nitrogen accumulation }\left({\text{kg ha}}^{-1}\right)=\text{soil nitrate nitrogen content }\left({\text{mg kg}}^{-1}\right)\times \text{soil thickness }\left(\text{cm}\right)\times \text{soil bulk density}\left({\text{g cm}}^{-3}\right)/10$$

According to the research of Li et al. (2025), the fuzzy mathematical membership function method was used to evaluate each fertilization practice comprehensively. If a certain index had a positive effect, X (u1) was used for calculation. If a certain index had a negative effect, X (u2) was used for calculation. According to the (Equations 1 or 2), the membership function value for each treatment was calculated, and then the average membership function value of each variety was calculated according to the (Equation 3).

(1) X(u1)=(X−Xmin)/(Xmax−Xmin)

(2) X(u2)=1−(X−Xmin)/(Xmax−Xmin)

In the formula, X(u) is the membership function value, X refers to the original measured value of an index, Xmin and Xmax are the minimum and maximum values of an index in each treatment, respectively.

(3) Xi=∑Xij/n

In the formula, Xi is the average membership function value, Xij is the jth index of the ith average membership function value, and n is the number of indexes to calculate the ith average membership function value.

The higher the mean membership function value, the better the overall performance.

## 3 Results

### 3.1 Effects of different fertilization practices on yield

The tomato yield under CK treatment was the lowest (74108 kg ha^-1^) (**Figure 1**). Yields from CF to MSRP+U treatments were 14.81% to 23.23% higher than that of CK. Among them, MSRP+U treatment resulted in the highest yield increase. However, there were no significant differences among OPN, SRUA+U, SRUB+U, and MSRP+U. Overall, tomato yield did not keep increasing with higher nitrogen application rates. The optimized nitrogen application in OPN treatment reduced nitrogen fertilizer input while increasing yield. In addition, compared with conventional farmer practice, the slow-release fertilizer treatment (SRUA+U, SRUB+U, and MSRP+U) significantly improved tomato yield.

**Figure 1:**
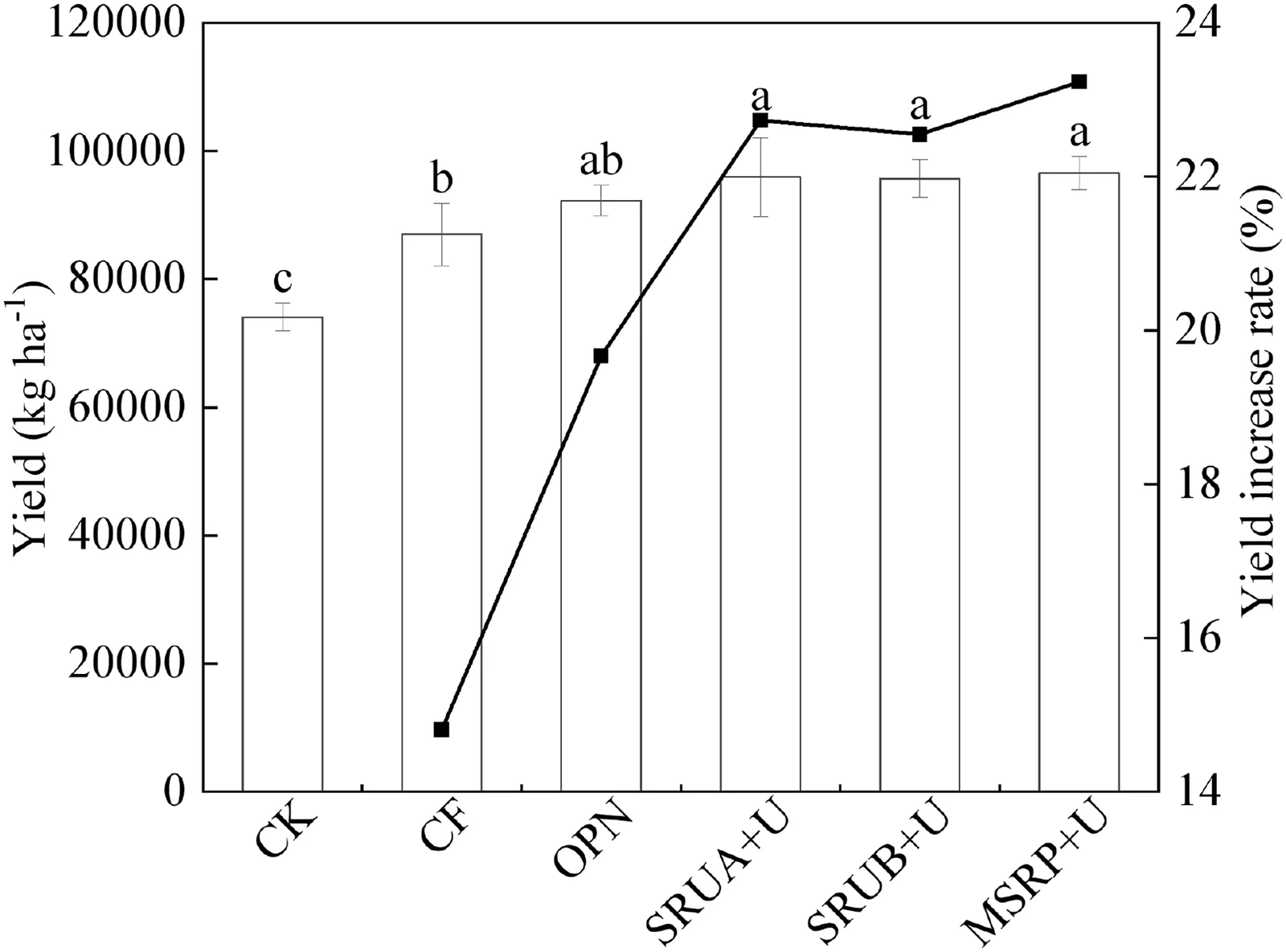
Effects of different fertilization practices on tomato yield (n=3). CK, (No nitrogen fertilizer), CF, (Conventional farmer practice, 360 kg ha^-1^ N), OPN, (Optimized nitrogen application, 240 kg ha^-1^ N), SRUA+U, (Slow-release urea A mixed with conventional urea at 7:3, 240 kg ha^-1^ N), SRUB+U, (Slow-release urea B mixed with conventional urea at 7:3, 240 kg ha^-1^ N), MSRP+U, (Conventional urea mixed with modified slow-release polymer material, 240 kg ha^-1^ N). Different lowercase letters indicate that there are significant differences at the level of 0.05. The error bars represent the mean ± SD.

### 3.2 Effects of different fertilization practices on quality

The Vitamin C content in treatments SRUA+U to MSRP+U was significantly higher than that in CK, with an increase of 17.10% to 20.57% (**Figure 2**). Although CF and OPN also had higher Vitamin C content than CK, the difference was not significant. All fertilization treatments had markedly higher soluble solids than the non-fertilization treatment (CK), by 17.42% to 25.10%. Among the optimized fertilization treatments, there was no significant difference in soluble solids content, but they were notably higher than the conventional farmer practice (CF) by 5.15% to 6.55%. The titratable acid content in CK and CF was significantly higher than in the other treatments, by 20.10%-28.63% and 11.13%-19.01%, respectively. However, there was no prominent difference among the optimized nitrogen treatments. As for the soluble sugar content, there was no significant difference among the optimized nitrogen treatments, but their values were dramatically higher than that of the no-nitrogen treatment, by 46.91% to 70.94%.

**Figure 2:**
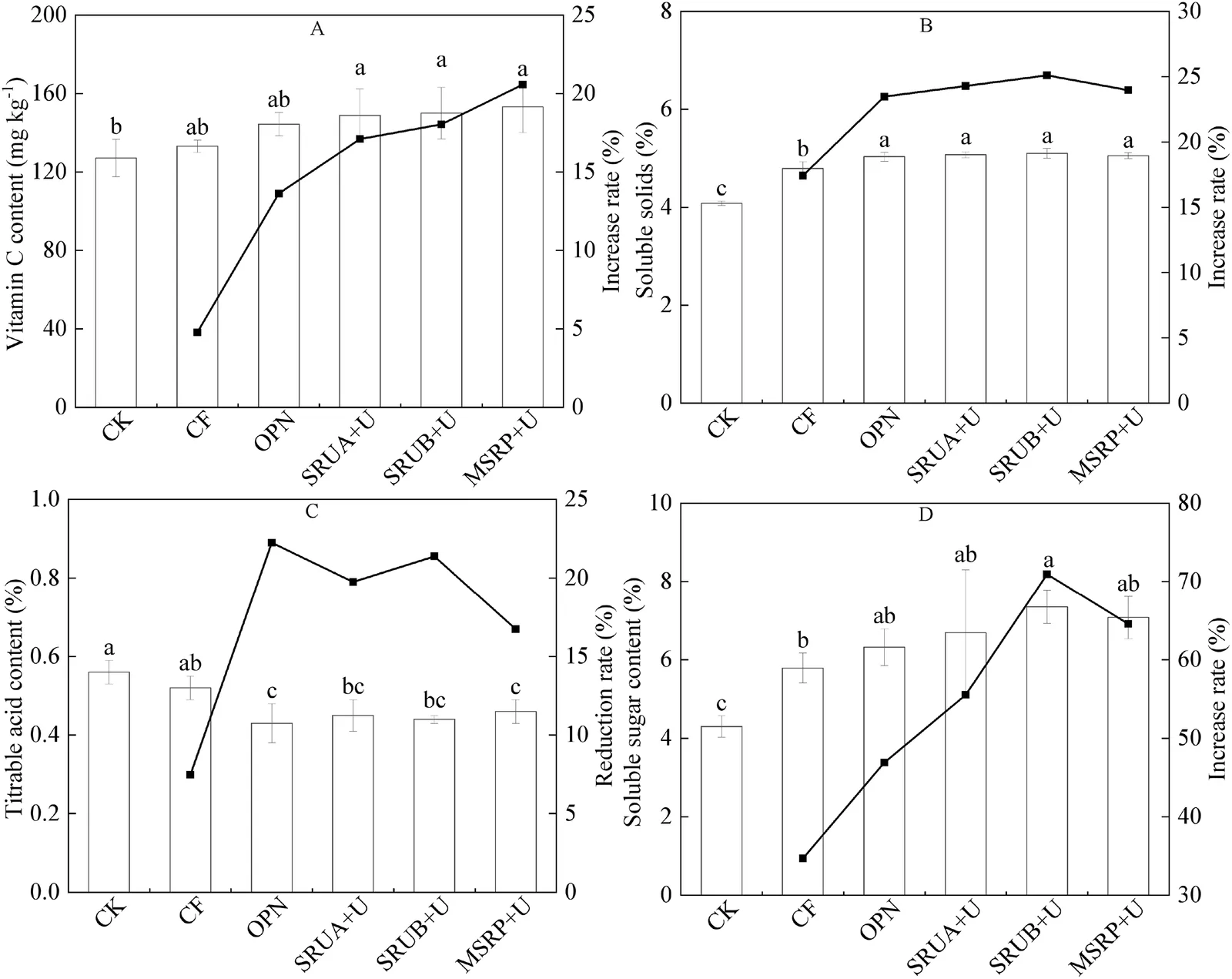
Effects of different fertilization practices on tomato quality (n=3). CK, (No nitrogen fertilizer), CF, (Conventional farmer practice, 360 kg ha^-1^ N), OPN, (Optimized nitrogen application, 240 kg ha^-1^ N), SRUA+U, (Slow-release urea A mixed with conventional urea at 7:3, 240 kg ha^-1^ N), SRUB+U, (Slow-release urea B mixed with conventional urea at 7:3, 240 kg ha^-1^ N), MSRP+U, (Conventional urea mixed with modified slow-release polymer material, 240 kg ha^-1^ N). Different lowercase letters indicate that there are significant differences at the level of 0.05. The error bars represent the mean ± SD. **(A)** Vitamin C content; **(B)** Soluble solids; **(C)** Titratable acid content; **(D)** Soluble sugar content.

### 3.3 Effects of different fertilization practices on agronomic traits

Plant height at flowering did not differ significantly among the SRUA+U, SRUB+U, and MSRP+U treatments (**Figure 3**). However, plant height in these treatments was significantly higher than in CK by 51.31%, 33.79%, and 50.69%, respectively. Stem diameter did not vary noticeably among treatments. The SPAD value was lowest in CK (45.40). It was significantly lower than those in SRUA+U, SRUB+U, and MSRP+U by 8.04%, 13.67%, and 13.47%, respectively. Among the nitrogen-applied treatments, SPAD values did not differ significantly.

**Figure 3:**
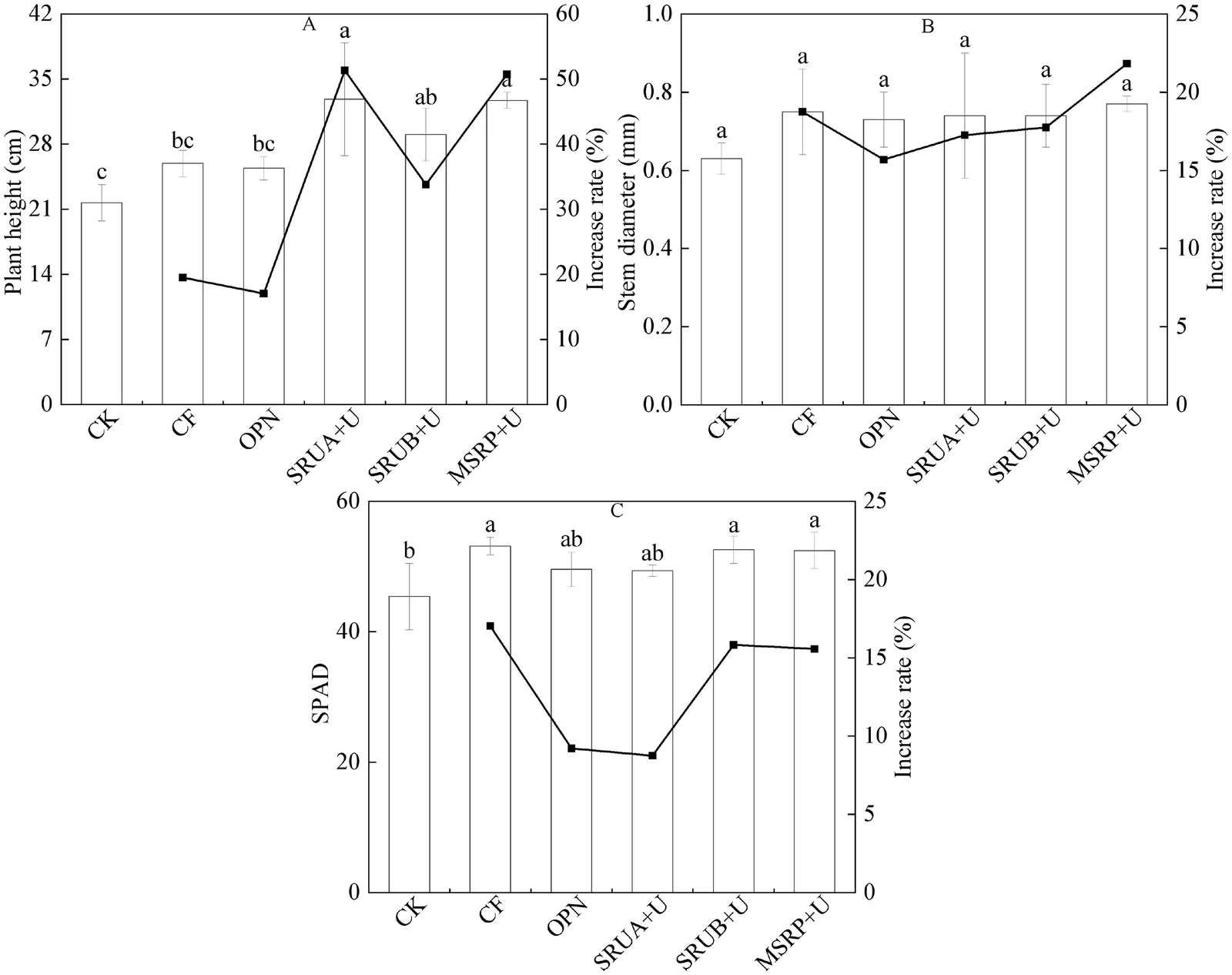
Effects of different fertilization practices on tomato agronomic traits (n=3). CK, (No nitrogen fertilizer), CF, (Conventional farmer practice, 360 kg ha^-1^ N), OPN, (Optimized nitrogen application, 240 kg ha^-1^ N), SRUA+U, (Slow-release urea A mixed with conventional urea at 7:3, 240 kg ha^-1^ N), SRUB+U, (Slow-release urea B mixed with conventional urea at 7:3, 240 kg ha^-1^ N), MSRP+U, (Conventional urea mixed with modified slow-release polymer material, 240 kg ha^-1^ N). Different lowercase letters indicate that there are significant differences at the level of 0.05. The error bars represent the mean ± SD. **(A)** Plant height; **(B)** Stem diameter; **(C)** SPAD.

### 3.4 Effects of different fertilization practices on soil nitrogen use efficiency

Fertilization practices significantly influenced the nitrogen agronomic efficiency and nitrogen fertilizer partial productivity (**Figure 4**). Among the optimized nitrogen application treatments (OPN, SRUA+U, SRUB+U, and MSRP+U), the nitrogen agronomic efficiency did not differ markedly. However, it was 171.31% to 231.95% higher than that of the conventional farmer practice (CF). A similar trend was observed for nitrogen fertilizer partial productivity. No significant differences were observed among the optimized treatments (OPN, SRUA+U, SRUB+U, and MSRP+U). However, all of these treatments showed significantly higher values than the conventional farmer practice (CF), with increases ranging from 59.08% to 66.45%. These results indicate that excessive nitrogen application reduces both the nitrogen agronomic efficiency and nitrogen fertilizer partial productivity.

**Figure 4:**
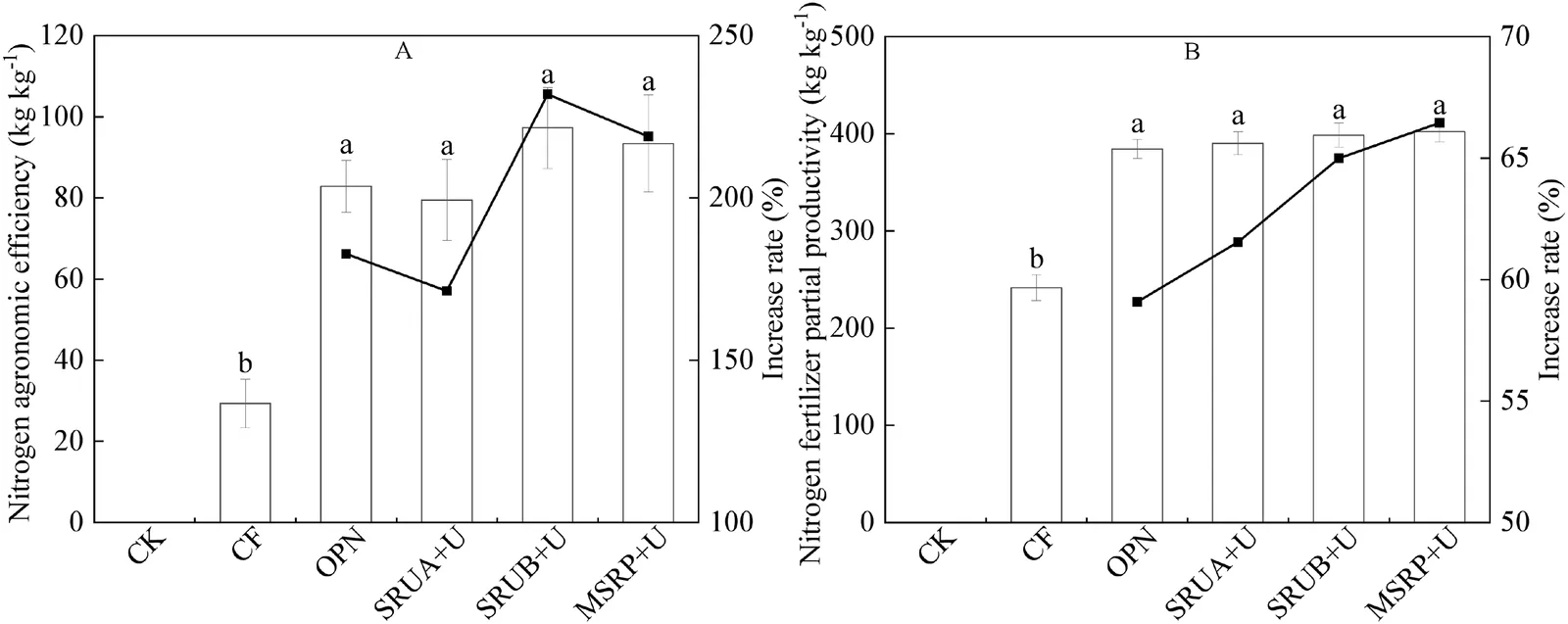
Effects of different fertilization practices on soil nitrogen use efficiency (n=3). CK, (No nitrogen fertilizer), CF, (Conventional farmer practice, 360 kg ha^-1^ N), OPN, (Optimized nitrogen application, 240 kg ha^-1^ N), SRUA+U, (Slow-release urea A mixed with conventional urea at 7:3, 240 kg ha^-1^ N), SRUB+U, (Slow-release urea B mixed with conventional urea at 7:3, 240 kg ha^-1^ N), MSRP+U, (Conventional urea mixed with modified slow-release polymer material, 240 kg ha^-1^ N). Different lowercase letters indicate that there are significant differences at the level of 0.05. The error bars represent the mean ± SD. **(A)** Nitrogen agronomic efficiency; **(B)** Nitrogen fertilizer partial productivity.

### 3.5 Effects of different fertilization practices on soil nitrate leaching

Different fertilization strategies significantly affect the profile distribution and leaching risk of soil nitrate nitrogen (**Figure 5**). With the development of the growth process, CF and OPN treatments maintained high nitrate concentrations in the topsoil, and the 0–20 cm soil layer increased by 72% and 67% respectively in the third fruiting stage compared with the first fruiting stage. However, in deep soil (40–100 cm), the content of nitrate nitrogen in CF and OPN treatments increased significantly in the late growth stage.

**Figure 5:**
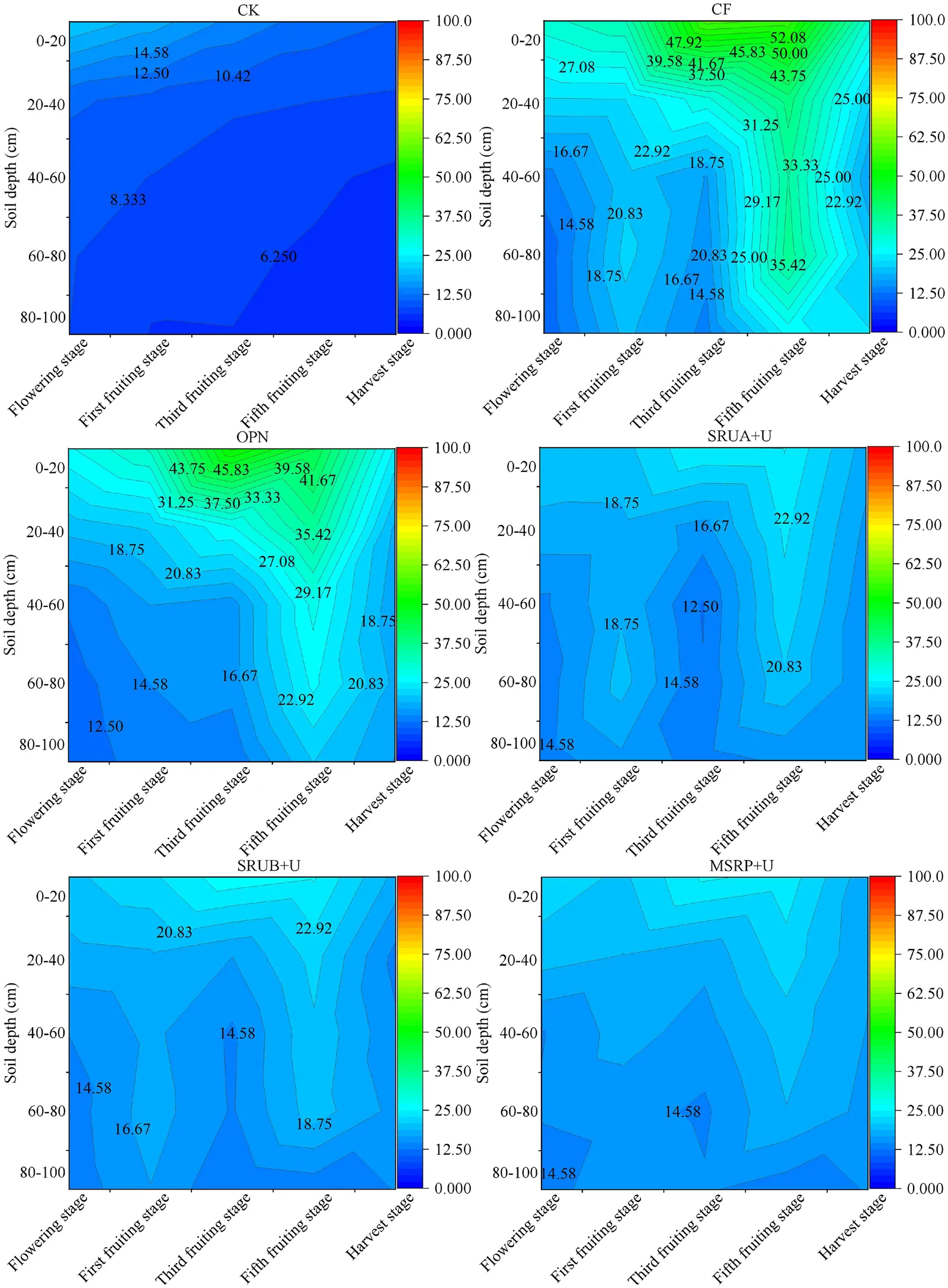
Effects of different fertilization strategies on temporal and spatial changes of nitrate nitrogen content in 0–100 cm soil layer (n=3). CK, (No nitrogen fertilizer), CF, (Conventional farmer practice, 360 kg ha^-1^ N), OPN, (Optimized nitrogen application, 240 kg ha^-1^ N), SRUA+U, (Slow-release urea A mixed with conventional urea at 7:3, 240 kg ha^-1^ N), SRUB+U, (Slow-release urea B mixed with conventional urea at 7:3, 240 kg ha^-1^ N), MSRP+U, (Conventional urea mixed with modified slow-release polymer material, 240 kg ha^-1^ N). Different lowercase letters indicate that there are significant differences at the level of 0.05. The error bars represent the mean ± SD.

The loss of nitrate nitrogen through leaching depends on the soil layer. According to the growth characteristics of tomato, the root system in facility-grown plants is mainly concentrated in the top 60 cm of soil. In the nutrient absorption layer of 0–60 cm tomato, the accumulation of nitrate nitrogen in CK treatment was significantly lower than that in other treatments, which could not meet the needs of plant growth and greatly limited the yield of tomato. However, CF treatment was significantly higher than other treatments by 13.12%-182.28%, resulting in a surplus of nitrogen, which could not be completely absorbed by aboveground plants, increasing the risk of nitrate leaching in 60–100 cm soil layer. However, the nitrate nitrogen content within the 0-60 cm soil layers remained relatively stable under slow and controlled release fertilizer treatments. When nitrate nitrogen leaches below 60 cm, it becomes difficult for tomato plants to absorb and utilize. Therefore, nitrate nitrogen accumulated in the 60–100 cm soil layer can be considered ineffective accumulation. The nitrate accumulation in the CK treatment was the lowest (29.94 kg ha^-1^), significantly lower than that in the fertilized treatments (64.35%-75.77%) (**Figure 6**). The conventional farmer practice had the highest accumulation (123.58 kg ha^-1^), which was significantly higher than that in the optimized nitrogen treatments by 25.99%-47.14%, indicating a high leaching risk. Among the optimized nitrogen treatments (SRUA+U, SRUB+U, and MSRP+U), there was no significant difference in nitrate nitrogen accumulation.

**Figure 6:**
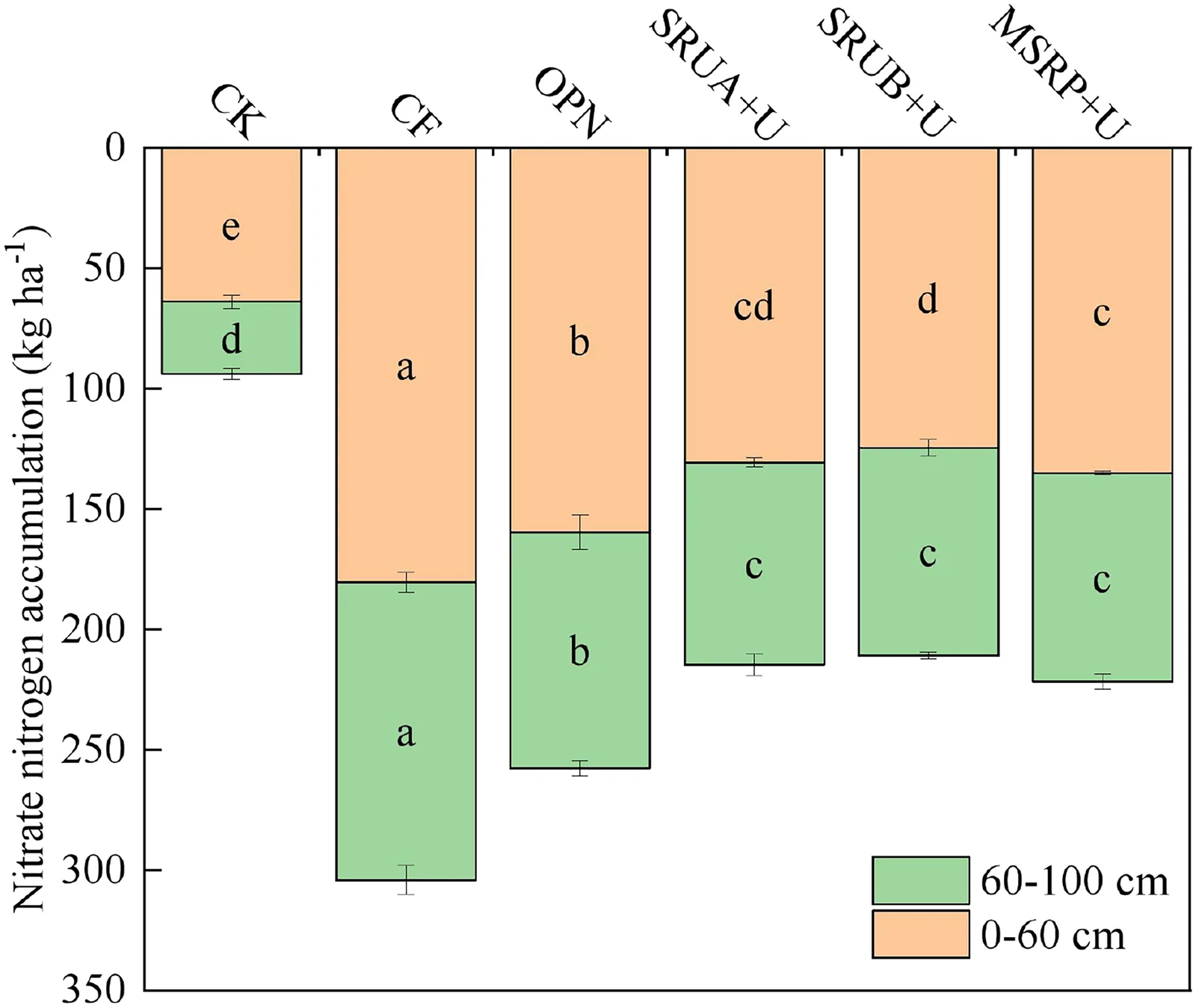
Effects of different fertilization practices on nitrate N accumulation in 0–100 cm soil layer (n=3). CK, (No nitrogen fertilizer), CF, (Conventional farmer practice, 360 kg ha^-1^ N), OPN, (Optimized nitrogen application, 240 kg ha^-1^ N), SRUA+U, (Slow-release urea A mixed with conventional urea at 7:3, 240 kg ha^-1^ N), SRUB+U, (Slow-release urea B mixed with conventional urea at 7:3, 240 kg ha^-1^ N), MSRP+U, (Conventional urea mixed with modified slow-release polymer material, 240 kg ha^-1^ N). Different lowercase letters indicate that there are significant differences at the level of 0.05. The error bars represent the mean ± SD.

### 3.6 Comprehensive evaluation of different fertilization practices

Evaluation based on a single index was not reliable. Therefore, a comprehensive assessment using multiple indicators was necessary to obtain more objective results. In this study, the membership function method was applied to evaluate the five fertilizer practices. The results indicated that the membership function values ranged from 0.15 to 0.91 (**Table 1**). Among the treatments, MSRP+U treatment performed the best, while CK performed the worst.

**Table 1:** Membership function values of different fertilization practices.

T	Y	VC	SS	TAC	SSC	NO_3_^--^-N	NAE	PFPN	PH	SD	SPAD	MFV	Rank
CF	0.00	0.00	0.00	0.04	0.00	0.00	0.00	0.00	0.07	0.52	1.00	0.15	5
OPN	0.55	0.56	0.78	0.96	0.64	0.64	0.79	0.89	0.00	0.04	0.06	0.51	4
SRUA+U	0.93	0.78	0.89	0.80	1.00	1.00	0.74	0.93	1.00	0.29	0.00	0.72	3
SRUB+U	0.91	0.84	1.00	0.91	0.94	0.94	1.00	0.98	0.49	0.37	0.86	0.84	2
MSRP+U	1.00	1.00	0.85	0.62	0.93	0.93	0.94	1.00	0.98	1.00	0.82	0.91	1

CF, (Conventional farmer practice, 360 kg ha^-1^ N), OPN, (Optimized nitrogen application, 240 kg ha^-1^ N), SRUA+U, (Slow-release urea A mixed with conventional urea at 7:3, 240 kg ha^-1^ N), SRUB+U, (Slow-release urea B mixed with conventional urea at 7:3, 240 kg ha^-1^ N), MSRP+U, (Conventional urea mixed with modified slow-release polymer material, 240 kg ha^-1^ N). T, treatment; Y, yield; VC, vitamin C content; SS, soluble solids; TAC, titratable acid content; SSC, soluble sugar content; PH, plant height; SD, stem diameter; MFV, membership function values.

## 4 Discussion

### 4.1 Effects of fertilization practices on greenhouse tomato yield and quality

Nitrogen most significantly influences tomato yield, quality, and fruit shape (Tei et al., 2025). According to Gao (2018), tomato plant growth improves with increasing nitrogen application up to a certain point. Beyond that point, excessive nitrogen can inhibit the growth of greenhouse tomatoes. Similarly, tomato yield increases with appropriate nitrogen fertilization but does not keep rising with further increases in nitrogen input. Xie et al. (2011) reported that tomato yield increased when nitrogen application was below 300 kg ha^-1^, but decreased when it exceeded this rate. In this study, plants receiving no nitrogen had the lowest plant height and SPAD values. All nitrogen treatments improved agronomic traits compared to the no-nitrogen control. However, CF treatment showed the poorest performance among all fertilization groups. In contrast, MSRP+U treatment led to better agronomic performance. These improvements contributed to higher yield and quality. Specifically, yields in treatments CF to MSRP+U increased by 14.81% to 23.23% compared to CK, with MSRP+U showing the greatest yield improvement. Overall, yield did not increase indefinitely with nitrogen application. The optimized nitrogen treatment (OPN) reduced fertilizer input while increasing yield. Compared to conventional farmer practice, slow-release fertilizers significantly enhanced tomato yield. In terms of fruit quality, excessive application of nitrogen fertilizer obviously reduced the quality. Cheng et al. (2021) concluded that the optimal nitrogen rate was between 236 and 354 kg ha^-1^ with the potential of significantly increasing tomato yield by 59.9%, enhancing Vitamin C, sugar/acid ratio, soluble sugar and total soluble solids by 18.8%, 33.5%, 23.5% and 11.9%, respectively, but decreasing lycopene by 10.8% and increasing nitrate content by 60.4%. Wu (2018) demonstrated that moderate nitrogen reduction could effectively improve tomato quality compared to conventional farmer practice. These findings show that natural soil nitrogen supply is limited in greenhouse tomato production in China. This is especially true in the cold regions of northwestern Hebei. Low spring temperatures reduce soil microbial activity and nitrogen mineralization (Maslov and Maslova, 2022), so soil nitrogen alone cannot support rapid tomato growth. However, excessive nitrogen application also limits yield and quality improvement (Zhao et al., 2022). Slow-release fertilizers offer advantages such as a long nutrient release period and reduced volatilization, leaching, and fixation (Rashid et al., 2021). They allow one-time base application while supplying nutrients throughout the growth cycle. This promotes bud differentiation, increases yield and efficiency, and improves the quality of eggplant and fruit crops. Moreover, they help maintain higher levels of available soil nutrients, supporting balanced plant development (Jiang, 2005).

### 4.2 Effects of fertilization practices on soil nitrate leaching

Nitrogen is an essential element for plant growth. It plays a vital role in plant life processes. Therefore, nitrogen fertilizer application deserves special attention in agricultural production (Elhanafi et al., 2019). Currently, many farmers use high fertilizer inputs to sustain high crop yields. However, nitrate nitrogen is not easily adsorbed by soil colloids. This increases the risk of leaching, leading to fertilizer loss and groundwater pollution (Hosen et al., 2026). In this experiment, conventional farmer practice led to the highest accumulation of nitrate nitrogen at 60–100 cm soil depth. This indicates a high risk of leaching into deeper soil layers. In contrast, optimized fertilization reduced nitrate nitrogen accumulation. Slow-release treatments were particularly effective in controlling nitrogen release. They maintained soil nitrate nitrogen at a relatively stable level (Liu et al., 2023). In particular, the treatment using modified slow-release polymer materials offered combined benefits. Under equal nitrogen input, it provided fast acting inorganic nitrogen, organic nitrogen, and controlled-release nitrogen together. It also allowed integration with water-fertilizer integration systems. This approach supported simplified production, improved nitrogen use efficiency, and reduced nitrate leaching without compromising yield or quality. These polymer materials, when used with fast-acting nitrogen, meet early crop nitrogen demand and reduce nitrate leaching during non-uptake periods (Li et al., 2024). In this study, five fertilization treatments were evaluated using the membership function method. The ranking from best to worst was: MSRP+U > SRUB+U > SRUA+U > OPN > CF. This result confirms that MSRP+U treatment performed best overall.

Commercially available slow-release urea fertilizers have been widely reported to mitigate nitrogen loss in protected vegetable systems (Vejan et al., 2021). Nevertheless, their nutrient release is largely temperature-dependent (Priya et al., 2024), which becomes a major bottleneck for cold-region solar greenhouse production with relatively low soil temperature in growing seasons. Consistent with this principle, our results showed that SRUA+U and SRUB+U partially reduced nitrate leaching but exhibited inferior comprehensive performance compared with MSRP+U under identical reduced N input. Different from conventional polymer amendments such as anionic polyacrylamide which mainly modify soil physical properties (Wu et al., 2011), our modified slow-release polymer material is a composite of polyacrylamide, polyvinyl alcohol and manganese sulfate that forms unique cross-linked network structures (Tian et al., 2019, 2020). The special hydrophilic network enables great water-holding capacity and, more importantly, modulates nitrogen release rhythm rather than merely improving soil water retention (Liu et al., 2024). This feature allows MSRP+U to synchronize nitrogen supply with tomato crop demand under low-temperature greenhouse conditions, stabilizing nitrate within the 0-60 cm root zone and reducing deep layer nitrate accumulation. Notably, our strategy mixes conventional urea with MSRP instead of adopting high-cost complete controlled release fertilizers. Combined with nitrogen reduction to 240 kg ha^-1^ N and compatibility with drip fertigation, this represents a practical low-cost technical option for cold region greenhouses in northern China. Our membership function further supported this point: MSRP+U achieved the highest comprehensive score by balancing tomato yield, fruit quality, nitrogen use efficiency and nitrate leaching risk, overcoming the common trade off dilemma between crop productivity and environmental risk in intensive greenhouse tomato production.

## 5 Conclusion

This study explored urea nitrogen effects on greenhouse tomato yield, quality, nitrogen use efficiency (NUE), and soil nitrate leaching under water-fertilizer integration in cold regions. Excessive conventional nitrogen application (360 kg ha^-1^ N) lowered NUE, failed to improve crop performance, and induced severe deep-soil nitrate leaching. In contrast, reduced nitrogen input (240 kg ha^-1^ N) effectively promoted tomato growth and quality while improving NUE and alleviating nitrate loss. Of the optimized treatments, the conventional urea mixed with modified slow-release polymer material (MSRP+U) produced the best comprehensive outcomes. This treatment concurrently maximized tomato yield and quality while minimizing nitrate leaching, as further validated by fuzzy membership function evaluation. In conclusion, the conventional urea mixed with modified slow-release polymer material constitutes an efficient, environment-friendly fertigation strategy suitable for cold-region greenhouses. It overcomes the low-temperature constraints of conventional slow-release fertilizers and adapts well to drip fertigation systems, providing a practical technique for sustainable high-quality tomato production in cold northern China.

## Data Availability

The data analyzed in this study is subject to the following licenses/restrictions: Data will be made available on request. Requests to access these datasets should be directed to Changqing LI, lixiaoqing@cdmc.edu.cn.

## References

[B1] Awad Y. M. Blagodatskaya E. Ok Y. S. Kuzyakov Y. (2012). Effects of polyacrylamide, biopolymer, and biochar on decomposition of soil organic matter and plant residues as determined by 14C and enzyme activities. Eur. J. Soil Biol. 48, 1–10. doi: 10.1016/j.ejsobi.2011.09.00542574925

[B2] Bao S. D. (2000). Soil and Agricultural Chemistry Analysis (Beijing: China Agriculture Press).

[B3] Chang L. Y. Xu L. J. Liu Y. H. Qiu D. (2021). Superabsorbent polymers used for agricultural water retention. Polym. Test. 94, 107021. doi: 10.1016/j.polymertesting.2020.10702142574925

[B4] Cheng M. Wang H. Fan J. Xiang Y. Zhang F. (2021). Effects of nitrogen supply on tomato yield, water use efficiency and fruit quality: A global meta-analysis. Sci. Hortic. 290, 110553. doi: 10.1016/j.scienta.2021.11055342574925

[B5] Elhanafi L. Houhou M. Rais C. Mansouri I. Elghadraoui L. Greche H. (2019). Impact of excessive nitrogen fertilization on the biochemical quality, phenolic compounds, and antioxidant power of Sesamum indicum L Seeds. J. Food Qual. 6, 9428092. doi: 10.1155/2019/9428092

[B6] Fertahi S. Ilsouk M. Zeroual Y. Oukarroum A. Barakat A. (2021). Recent trends in organic coating based on biopolymers and biomass for controlled and slow release fertilizers. J. Control Release 330, 341–361. doi: 10.1016/j.jconrel.2020.12.026 33352245

[B7] Gao J. F. (2006). Experimental Guidance on Plant Physiology (Beijing: Higher Education Press).

[B8] Gao X. S. (2018). Effects of different nitrogen levels on growth and development of greenhouse tomato. Mod. Agric. Sci. Technol. 21, 60–62. doi: 10.3969/j.issn.1007-5739.2018.21.038

[B9] Govil S. Long N. V. D. Escrib`a-Gelonch M. Hessel V. (2024). Controlled-release fertiliser: Recent developments and perspectives. Ind. Crop Prod. 219, 119160. doi: 10.1016/j.indcrop.2024.11916042574925

[B10] Hosen M. H. A. Das S. Burns G. L. Chaki A. K. Neogi M. G. Rahman M. W. . (2026). Nitrogen fertilizer use in agroecosystems: current practices, policies and recommendations for efficiency improvement. J. Soils Sediments 26, 225. doi: 10.1007/s11368-026-04423-530311153

[B11] Jariwala H. Santos R. M. Lauzon J. D. Dutta A. Chiang Y. W. (2022). Controlled release fertilizers (CRFs) for climate-smart agriculture practices: a comprehensive review on release mechanism, materials, methods of preparation, and effect on environmental parameters. Environ. Sci. pollut. Res. 29, 53967–55399. doi: 10.1007/s11356-022-20890-y 35624378

[B12] Jiang B. L. (2005). Effects of Contolled-Release Fertilizers on Dynamic Changes of Nutrient and Fruit Quality of Vegetable of Nightshade (Taian: Shandong Agricultural University). [dissertation/master’s thesis].

[B13] Lee S. S. Gantzer C. J. Thompson A. L. Anderson S. H. (2010). Polyacrylamide and gypsum amendments for erosion and runoff control on two soil series. J. Soil Water Conserv. 65, 233–242. doi: 10.2489/jswc.65.4.233

[B14] Li Y. Niu W. Q. Dyck M. Wang J. W. Zou X. Y. (2016). Yields and nutritional of greenhouse tomato in response to different soil aeration volume at two depths of subsurface drip irrigation. Sci. Rep. 6, 39307. doi: 10.1038/srep39307 27995970 PMC5171848

[B15] Li Y. M. Sun Y. X. Liao S. Q. Zou G. Y. Zhao T. K. Chen Y. H. . (2017). Effects of two slow-release nitrogen fertilizers and irrigation on yield, quality, and water-fertilizer productivity of greenhouse Tomato. Agr Water Manage. 186, 139–146. doi: 10.1016/j.agwat.2017.02.00642574925

[B16] Li C. Q. Tong B. X. Jia M. Y. Xu H. S. Wang J. Q. Sun Z. M. (2024). Integrated management strategies increased silage maize yield and quality with lower nitrogen losses in cold regions. Front. Plant Sci. 15, 1434926. doi: 10.3389/fpls.2024.1434926 39104846 PMC11298381

[B17] Li C. Y. Zhang R. R. Zhu H. Y. You D. M. Hu H. Y. Li Z. Z. . (2025). Effects of salt stress on physiological characteristics of alfalfa seedlings and evaluation of salt tolerance at seedling stage. Acta Agrestia Sin. 33, 516–523. doi: 10.11733/j.issn.1007-0435.2025.02.021

[B18] Lin X. Deng Z. P. Yang X. Y. Zhou K. Y. Yi X. P. Wang J. C. (2025). Effects of reducing chemical fertilizer combined with organic fertilizer on potato yield and nutrient utilization. Acta Hortic. Sin. 52, 1007–1019. doi: 10.16420/J.ISSN.0513-353X.2023-1022

[B19] Lin X. Y. Guo L. Z. Shaghaleh H. Hamoud Y. A. Xu X. Liu H. (2021). A TEMPO-oxidized cellulose nanofibers/MOFs hydrogel with temperature and pH responsiveness for fertilizers slow-release. Int. J. Biol. Macromol. 191, 483–491. doi: 10.1016/j.ijbiomac.2021.09.075 34562535

[B20] Liu H. L. Lei F. Li D. Yang H. Z. Luo W. Zhu Z. Q. . (2023). Evaluation of nitrogen release characteristics and enhanced efficiency of a novel synthetic slow-release nitrogen fertilizer. J. Soil Sci. Plant Nutr. 23, 5671–5682. doi: 10.1007/s42729-023-01432-y30311153

[B21] Liu M. S. Li J. X. Ren B. C. Liu Y. Liu Z. H. Zhou T. T. . (2024). The water-retaining functional slow-release fertilizer modified by carboxymethyl chitosan. Carbohydr. Polym. 328, 121744. doi: 10.1016/j.carbpol.2023.121744 38220354

[B22] Liu R. L. Wang Y. Hong Y. Wang F. Mao X. P. Yi J. (2023). Controlled-release urea application and optimized nitrogen applied strategy reduced nitrogen leaching and maintained grain yield of paddy fields in Northwest China. Front. Plant Sci. 14, 1033506. doi: 10.3389/fpls.2023.1033506 36778679 PMC9909425

[B23] Lu S. Y. Chen F. X. Ngo H. H. Guo W. S. Feng C. P. Wu J. N. . (2016). Effect of straw and polyacrylamide on the stability of land/water ecotone soil and the field implementation. Ecol. Eng. 94, 12–21. doi: 10.1016/j.ecoleng.2016.05.07642574925

[B24] Luo J. Q. Wang L. L. Li Q. S. Zhang Q. K. He B. Y. Wang Y. . (2015). Improvement of hard saline-sodic soils using polymeric aluminum ferric sulfate (PAFS). Soil Tillage Res. 149, 12–20. doi: 10.1016/j.still.2014.12.01442574925

[B25] Maslov M. N. Maslova O. A. (2022). Soil nitrogen mineralization and its sensitivity to temperature and moisture in temperate peatlands under different land-use management practices. Catena 210, 105922. doi: 10.1016/j.catena.2021.10592242574925

[B26] Padayachee A. Day L. Howell K. Gidley M. J. (2017). Complexity and health functionality of plant cell wall fibers from fruits and vegetables. Crit. Rev. Food Sci. Nutr. 57, 59–81. doi: 10.1080/10408398.2013.850652 25830345

[B27] Priya E. Sudipta S. Maji P. K. (2024). A review on slow-release fertilizer: Nutrient release mechanism and agricultural sustainability. J. Environ. Chem. Eng. 12, 113211. doi: 10.1016/j.jece.2024.11321142574925

[B28] Ransom C. J. Jolley V. D. Blair T. A. Sutton L. E. Hopkins B. G. (2020). Nitrogen release rates from slow and controlled-release fertilizers influenced by placement and temperature. PloS One 15, e0234544. doi: 10.1371/journal.pone.0234544 32555670 PMC7299380

[B29] Rashid M. Hussain Q. Khan K. S. Alwabel M. I. Hayat R. Akmal M. . (2021). Carbon-based slow-release fertilizers for efficient nutrient management: synthesis, applications, and future research needs. J. Soil Sci. Plant Nutr. 21, 1144–1169. doi: 10.1007/s42729-021-00429-930311153

[B30] Rathore A. C. Mehta H. Jayaprakash J. Singh C. Gupta A. K. Kumar P. . (2021). Modified plant architecture integrated with liquid fertilizers improves fruit productivity and quality of tomato in North West Himalaya, India. Sci. Rep. 11, 18664. doi: 10.1038/s41598-021-98209-z 34545161 PMC8452768

[B31] Rop K. Karuku G. N. Mbui D. Michira I. Njomo N. (2018). Formulation of slow release NPK fertilizer (cellulose-graft-poly(acrylamide)/nano-hydroxyapatite/soluble fertilizer) composite and evaluating its N mineralization potential. Ann. Agric. Sci. 63, 163–172. doi: 10.1016/j.aoas.2018.11.00142574925

[B32] Sha Z. P. Ma X. Wang J. X. Lv T. T. Li Q. Q. Misselbrook T. . (2020). Effect of N stabilizers on fertilizer-N fate in the soil-crop system: A meta-analysis. Agr Ecosyst. Environ. 290, 106763. doi: 10.1016/j.agee.2019.10676342574925

[B33] Sojka R. E. Bjorneberg D. L. Entry J. A. Lentz R. D. Orts W. J. (2007). Polyacrylamide in agriculture and environmental land management. Adv. Agron. 92, 75–162. doi: 10.1016/S0065-2113(04)92002-0

[B34] Tei F. Benincasa P. Falcinelli B. Guiducci M. Onofri A. Pannacci E. . (2025). Nitrogen management in processing tomato: An overview. Ital. J. Agron. 20, 100069. doi: 10.1016/j.ijagro.2025.10006942574925

[B35] Tian X. M. Fan H. Wang J. Q. Ippolito J. Li Y. B. Feng S. S. . (2019). Effect of polymer materials on soil structure and organic carbon under drip irrigation. Geoderma 340, 94–103. doi: 10.1016/j.geoderma.2018.12.03842574925

[B36] Tian X. M. Wang K. Y. Liu Y. H. Liu Y. H. Fan H. Wang J. Q. . (2020). Effects of polymer materials on soil physicochemical properties and bacterial community structure under drip irrigation. Appl. Soil Ecol. 150, 1–9. doi: 10.1016/j.apsoil.2019.10345642574925

[B37] Vejan P. Khadiran T. Abdullah R. Ahmad N. (2021). Controlled release fertilizer: A review on developments, applications and potential in agriculture. J. Controlled Release 339, 321–334. doi: 10.1016/j.jconrel.2021.10.003 34626724

[B38] Wu X. Y. (2018). Effects of Nitrogen Fertilizer Reduction on Yield and Quality of Greenhouse Tomato (Yangling: Northwest Agriculture and Forestry University). [dissertation/master’s thesis].

[B39] Wu L. Ok Y. S. Xu X. L. Kuzyakov Y. (2011). Effects of anionic polyacrylamide on maize growth: A short term 14C labeling study. Plant Soil 350, 311–322. doi: 10.1007/s11104-011-0911-y30311153

[B40] Xie A. K. Li Z. H. Zhang Y. G. Zhang G. L. Wu C. F. Sun J. . (2011). Effect of different application of nitrogen on yield and quality of tomato and nitrate in soil profile. SFSiC 1, 26–29+68. doi: 10.3969/j.issn.1673-6257.2011.01.006

[B41] Xing F. W. Li X. T. Liu W. Chen Y. J. Zhang J. X. Long B. L. . (2026). Integration of water-retaining polymers into cemented soil to revegetate steep rock-cut slopes in hydropower projects. Mater. Today Sustain. 34, 101338. doi: 10.1016/j.mtsust.2026.10133842574925

[B42] Zhao C. Liu G. M. Chen Y. Jiang Y. Shi Y. Zhao L. T. . (2022). Excessive nitrogen application leads to lower rice yield and grain quality by inhibiting the grain filling of inferior grains. Agriculture 12, 962. doi: 10.3390/agriculture1207096230654563

